# Tolerance to sustained activation of the cAMP/Creb pathway activity in osteoblastic cells is enabled by loss of p53

**DOI:** 10.1038/s41419-018-0944-8

**Published:** 2018-08-28

**Authors:** Mannu K. Walia, Scott Taylor, Patricia W. M. Ho, T. John Martin, Carl R. Walkley

**Affiliations:** 1 St. Vincent’s Institute of Medical Research, Fitzroy, VIC 3065 Australia; 2 Department of Medicine, St Vincent’s Hospital, University of Melbourne, Fitzroy, VIC 3065 Australia; 3 Mary MacKillop Institute for Health Research, Australian Catholic University, Melbourne, VIC 3000 Australia

## Abstract

The loss of p53 function is a central event in the genesis of osteosarcoma (OS). How mutation of p53 enables OS development from osteoblastic lineage cells is poorly understood. We and others have reported a key role for elevated and persistent activation of the cAMP/PKA/Creb1 pathway in maintenance of OS. In view of the osteoblast lineage being the cell of origin of OS, we sought to determine how these pathways interact within the context of the normal osteoblast. Normal osteoblasts (p53 WT) rapidly underwent apoptosis in response to acute elevation of cAMP levels or activity, whereas p53-deficient osteoblasts tolerated this aberrant cAMP/Creb level and activity. Using the p53 activating small-molecule Nutlin-3a and cAMP/Creb1 activator forskolin, we addressed the question of how p53 responds to the activation of cAMP. We observed that p53 acts dominantly to protect cells from excessive cAMP accumulation. We identify a Creb1-Cbp complex that functions together with and interacts with p53. Finally, translating these results we find that a selective small-molecule inhibitor of the Creb1-Cbp interaction demonstrates selective toxicity to OS cells where this pathway is constitutively active. This highlights the cAMP/Creb axis as a potentially actionable therapeutic vulnerability in p53-deficient tumors such as OS. These results define a mechanism through which p53 protects normal osteoblasts from excessive or abnormal cAMP accumulation, which becomes fundamentally compromised in OS.

## Introduction

Osteosarcoma (OS) is the most common cancer of bone and primarily affects teenagers and young adults. Whilst our understanding of the genetics of OS have rapidly advanced, clinical outcomes have stagnated for several decades. OS is a cancer with many complex genetic abnormalities, but few genetic drivers. Conventional human OS has very high, to near universal, rates of *TRP53* mutation with recurrent mutations of *RB1*, *ATRX*, and *DLG2* in 29–53% of cases^1–3^. Genome-wide association studies *(GWAS)* and sequencing studies have identified mutations in key components of the cAMP pathway within the mutational spectrum of human OS^4,5^. Several recent studies in murine models have provided further evidence for involvement of the cAMP-PKA pathway in OS^6–9^, but how these pathways interact in the normal osteoblasts has not been resolved.

During normal bone development, osteoblastic lineage cells communicate, regulate, and activate each other through the secretion of specific molecules including parathyroid hormone-related protein (PTHrP). PTHrP acts through its cell surface receptor PTHR1, with evidence also for an intracrine action^10,11^. Osteoblast-specific ablation of *Pthrp* in mice resulted in impaired bone formation both in vivo and ex vivo^12,13^. These findings identified a central role for osteoblast lineage generated PTHrP in the physiological regulation of bone formation. This paracrine role was later extended when PTHrP production by osteocytes was found to be necessary for normal bone formation and strength^14^. As osteoblastic cells commit to form mature osteoblasts and ultimately osteocytes, PTHR1 expression increases and so does signaling via PTHrP^14^. PTHR1/PTHrP acts primarily to activate adenylyl cyclase and stimulate cAMP production^15^. Primary tumor cell cultures from mouse models of OS demonstrate both elevated and persistently active cAMP signaling, significantly contributed to by an autocrine PTHR1-PTHrP loop^6,8,16^.

Inactivating *TRP53* mutations are one of the most common mutations in human cancer^17^. The most frequent mutation type is point mutation resulting in P53 proteins with altered function^18^. Unlike most cancers, in OS unique genomic rearrangements and other mutation types often result in null alleles of *TRP53*^1,3^, and *TRP53* is the most recurrently mutated gene in OS^1^. *TRP53* mutations are also hallmark of the hereditary cancer predisposition disorder Li-Fraumeni syndrome^3,7,19,20^, and *Tp53* knockout mice develop OS at high penetrance amongst other tumors^21,22^. P53 is activated upon genotoxic or oncogenic stress and regulates cell cycle, survival, and apoptosis^23–29^. P53 also regulates “non-canonical” programs such as differentiation, autophagy, metabolism, cellular pluripotency, and plasticity^30^. P53 can mediate its non-canonical action via its interaction with a large number of transcription factors and coactivators^31^. Of relevance to OS, P53 regulates osteoblastic differentiation and *Tp53*^−/−^ mice have altered osteoblast differentiation and a high bone mass phenotype^32^.

In osteoblastic cells, activation of the cAMP pathway ultimately leads to activation of the transcription factor Creb1. Creb1 activates transcription of many cellular genes in response to cAMP. Numerous reports describe the role of CREB1 in malignancies such as AML, prostate cancer and other solid tumors including OS^8,33–35^. When phosphorylated on serine 133, CREB1 can facilitate the interaction between CBP and P53 on P53-responsive promoters^36^. We recently described the interaction of the cAMP/Creb1 pathway with p53 deletion in the maintenance of OS^8^. Herein, we have explored the interaction between these pathways in normal osteoblasts. We find that p53 normally acts to protect osteoblasts from aberrantly elevated cAMP/Creb activity, and that activation of this pathway can be tolerated once p53 is lost. Moreover, this dependence reveals a new therapeutic vulnerability in OS cells.

## Materials and methods

### Study approval

All animal experiments were approved by the AEC (AEC#030/14 and AEC#031/15; St. Vincent’s Hospital, Melbourne).

### Animals and cell culture

Long-bone primary osteoblastic cells and mouse OS cell cultures were cultured in αMEM (Lonza), 10% non-heat inactivated FBS (SAFC Biosciences) and 1% Penicillin/Streptomycin/Glutamine (Life Technologies). OS cell cultures were derived by mincing tumor tissue with a scalpel. The resulting tumor cells were transferred to a 6 well plate and allowed to establish in standard culture conditions. Primary cultures were passaged at 60–80% confluence with media changes every 2–3 days. The primary osteoblastic cells were derived from long-bone tissue, cleaned, and crushed lightly with a mortar-pestle. The suspension was rinsed with PBS, filtered to remove the majority of the hematopoietic (solution was clear). The bone fragments were placed in 15 ml of collagenase I (3 mg/ml) (Worthington), and incubated in a shaking 37 °C water bath for 45 min. A volume of 35 ml of PBS/2% FBS was then added and the cell suspension was sieved. The resulting population of long-bone derived cells was centrifuged at 400× *g* for 5 min, the cells were resuspended in culture media and plated onto a 6-well plate. On the next day, the 6-well plate was washed with PBS before adding fresh culture media to remove floating debris. At 48 h post-derivation, the cells were used for experiments.

### Generation of isogenic p53^WT/WT^ and p53^KO/KO^ normal osteoblastic cells

For experiments involving *R26*-CreER^T2ki/+^*p53*^*fl/fl*^, primary osteoblasts were isolated from long bones using collagenase digestion protocol as above. After 48 h the cells were split into two separate T25cm^2^ flask containing α-MEM (Lonza, Basel, Switzerland) supplemented with 10% fetal bovine serum (Sigma, St. Louis, MO, USA; non-heat inactivated), 2 mM Glutamax (Life Technologies, Carlsbad, CA, USA). The cells were treated with and without 500 nM 4-hydroxy-tamoxifen (Merck Millipore) to induce CRE activity and deletion of *p53* over 21 days. Deletion of p53 was confirmed by genomic DNA PCR and western blotting in the KO cultures treated with tamoxifen, compared to non-tamoxifen treated isogenic *R26*-CreER^T2ki/+^*p53*^*fl/fl*^ cultures. Three independently derived *R26*-CreER^T2ki/+^*p53*^*fl/fl*^ cultures were generated and used for experiments.

### OS cells

Primary mouse OS cell cultures were derived from primary tumors from murine models of fibroblastic OS (*Osx-*Cre *p53*^*fl/fl*^*pRb*^*fl/fl*^) or osteoblastic OS (*Osx*-Cre Tgshp53.1224*pRb*^*fl/fl*^) and were maintained and studied for less than 15 passages^16,22^. All cells used were obtained directly from mouse models of OS, no further authentication performed by the authors, mycoplasma negative.

### In vitro differentiation of p53^WT/WT^ and p53^KO/KO^ cells

Three independently derived *p53*^*WT/WT*^ and *p53*^*KO/KO*^ *cells* were seeded at 3000 cells/cm^2^ on 6-well plates in α-MEM with 10% FBS three days prior to differentiation induction. When cells had reached 100% confluence (Day 0), control cells were harvested, and all other cells were replenished three times per week with osteoblastic differentiation media: α-MEM (Lonza), 10% (v/v) FBS, 25 mM HEPES, 1% (v/v) (Gibco), Penicillin-Streptomycin (Gibco), 2 mM GlutaMAX™ (Gibco), 50 μg/ml ascorbate (Sigma), 0.01 M β-glycerophosphate (Sigma).

### IBMX treatment and cAMP assays

*p53*^*WT/WT*^ and *p53*^*KO/KO*^ *cells* were seeded at 1.0 × 10^5^ cells on 6-well plates in α-MEM with 10% FBS 1 day prior to IBMX treatment. 1 mM IBMX was added to the medium and the cells were incubated for 1 h before taking images. The cells were further incubated for 16–18 h before extracting lysates for western blotting. cAMP assay was performed as described previously^6,8^.

### Radioimmunoassay (RIA) for PTHrP

After the cells became confluent, fresh α-MEM (Lonza), 10% (v/v) FBS, 25 mM HEPES, 1% Penicillin-Streptomycin (Gibco), 2 mM GlutaMAX™ (Gibco). The medium was aspirated and the cells were washed once with PBS. RIA was carried out using polyclonal goat antiserum raised in goats against PTHrP(1–40), using recombinant PTHrP(1–84) as standard^6^. The sensitivity of this assay is 2pmol/l.

### PI staining

Adherent cells were washed in PBS, trypsinised, and pooled with the dead population of the same well. The cells were counted, resuspended in PBS and approximately 500,000 cells were transferred to a V-bottom plate. The plate was centrifuged, the cell pellet was dispersed and 100–150 μl of −20 °C chilled Ethanol was added to the cells. After brief incubation on ice, cells were pelleted, the ethanol was removed, and the cells were washed in PBS. Finally, the cells were resuspended in 100 μl PBS containing 40 μg/ml PI (Sigma-Aldrich, MO, USA) and 40 μg/ml RNAseA (Qiagen). Following PI addition, the cells were incubated in the dark for 30–60 min at 37 °C and analysed using a Fortessa FACS analyser (BD). The data were further analysed using flow cytometry analysis software FlowJo and the DNA cycles were determined using software Modfit Lt^TM^.

### Plasmids and constructs

Two independent *p53*^*WT/WT*^ cell lines were used for overexpressing pLenti6/V5-p53_wt p53 construct, a gift from Bernard Futscher (Addgene plasmid # 22945^37^). Lentiviral packaging vector psPax2 (plasmid #12260) was obtained from Addgene (Cambridge, MA, USA), the pCMV-Eco Envelope (Cat No. RV112) vector was purchased from Cell Biolabs (San Diego, CA, USA). Two independent isogenic *p53*^*WT/WT*^ cell lines were infected with lentivirus overexpressing human V5 tagged-P53. After 48 h cells were selected with 10 µg/ml Blasticidine S. The cells were confirmed for overexpression of WT V5-p53 by western blot analysis and used for experiments. For the construction of plasmid encoding a mouse p53 transcript, a cDNA encoding the mouse p53 transcript (IDT DNA) was cloned in pLVX vector (Clontech). Three independent Trp53^*WT/WT*^ cell lines were used for overexpressing pLVX-p53_wt p53 construct. The cells were confirmed for overexpression of p53 by western blot analysis and qPCR and used for experiments.

### Flow cytometry

Three independent isogenic *p53*^*WT/WT*^ and *p53*^*KO/KO*^ cell lines were prepared by trypsinization. Antibodies against murine CD45, Mac1, Gr1, F4/80, B220, IgM, CD2, CD3, CD4, CD8, Ter119, Sca1, CD51, PDGFRα (CD140a), CD31, either biotinylated or conjugated with eF450, PE, PerCP-Cy5.5, or APC were obtained from eBioscience (San Diego, CA) or BD Pharmingen. Biotinylated antibodies were detected with Streptavidin-Qdot605 (Invitrogen). Flow cytometry was performed on an LSRII Fortessa (BD Bioscience) interfaced with Cell Quest software, data were analysed on FlowJo (TreeStar).

### Annexin V and 7AAD staining

Three independent isogenic *p53*^*WT/WT*^ and *p53*^*KO/KO*^ cell lines were treated with 1 mM IBMX and 10 µM Forskolin for 1 h and 24 h, respectively. Cells were washed and collected after trypsinization and then stained in 1× Annexin Binding buffer (eBioscience) diluted 1:20 with Annexin V-APC (1 mg/ml) (eBioscience) and 7-Aminoactinomycin D (7AAD) (100 μg/ml) (Life Technologies) for 15 min. Following the addition of 4 volumes of 1× Annexin Binding buffer, apoptotic cells were detected and quantified using FACS (LSRFortessa). Live cells (Annexin V negative, 7AAD low) and cells in early and late stages of apoptosis (Annexin V positive, 7AAD low/high) were quantified.

### RNA extraction, cDNA synthesis and Quantitative realtime PCR (QPCR)

RNA was extracted using RNA extraction kits with on-column DNase digestion (Qiagen, Limburg, Netherlands; Bioline, London, UK) or TriSure reagent (Bioline). cDNA was synthesised from total RNA using a Tetro cDNA synthesis kit (Bioline) or AffinityScript cDNA synthesis kit (Agilent Technologies, Santa Clara, CA, USA). Gene expression was quantified on a Stratagene Mx3000P QPCR system (Agilent) using Brilliant II SYBR green QPCR master mix (Agilent) with primers specific to genes of interest (Primer sequences in Table S1). Relative expression was quantified using the comparative CT method (2^-(Gene Ct – Normalizer Ct)^). Samples were amplified in duplicate.

### Western blotting

Protein lysates were prepared in RIPA buffer (50 mM Tris-HCl pH7.4, 1% NP-40, 0.5% sodium deoxycholate, 0.1% SDS, 150 mM NaCl, 2 mM EDTA, 50 mM NaF). Protein (10–25 µg) was electrophoresed on 10% Bis Tris or 4–12% Bis-Tris gradient NuPAGE Novex protein gels (Life Technologies) and transferred to PVDF membrane (Merck Millipore, Billerica, MA, USA). Membranes were blocked in 5% skim milk in TBST (20 mM Tris, 150 mM NaCl, 0.1% Tween-20) for 1 h before incubation with primary antibodies diluted in 5% skim milk in TBST overnight a 4 °C, or for 1 h at room temperature in the case of pan-ACTIN. All antibodies, p53 (1C12; Cell Signaling 2524), Phospho-CREB ((Ser133) Cell Signaling Technologies, #9198), Anti-CREB1 ChIP grade (ab31387), and CBP (C-1, Santa Cruz, sc-7300) were used at 1:2000, except pan-actin (Ab-5, Thermo Scientific, Waltham, MA, USA) that was used at 1:3000. Cleaved caspase 3 Asp175 [5A1E] was from Cell Signaling Technologies 9664 S. Following four 10 min washes with TBST, membranes were exposed to ECL Prime (GE Healthcare Life Sciences, Piscataway, NJ, USA) and exposed to X-ray film to detect the expression levels of proteins.

### Immunoprecipitation

1 × 10^6^ cells for each subtype (Three independent isogenic *p53*^*WT/WT*^ and *p53*^*KO/KO*^) were seeded and allowed to proliferate for 24 h. *p53*^*WT/WT*^ and *p53*^*KO/KO*^ cells were treated with less than 0.1% DMSO, 10 µM forskolin or 100 nM doxorubicin and in combination. Cells were also treated with 0.1% DMSO, 10 µM forskolin or 10 µM Nutlin and in combination). Post treatment the cells were scraped on ice cold PBS and snap frozen until further use. For immunoprecipitation, non-ionic lysis buffer containing 1% NP-40, Tris-HCl at pH8.0, 137 mM NaCl and 2 mM EDTA with protease inhibitors (Roche, Burlington, NC, USA) was used. 2–3 × 10^6^ million cells were lysed in non-ionic lysis buffer and left on ice for 30 min, and the cells were further sonicated using a UCD-200 Bioruptor (Diagnenode, Denville, NJ, USA) on high at 4 °C for a total shearing time of 2 min (60 min of 10 s on and 50 s off). Cell debris was cleared by centrifugation at 13,000 rpm for 10 min at 4 °C and supernatants were diluted 10-fold in 1:10 diluted non-ionic lysis buffer. After removing 1% input for the total number of cells of each sample as an input control, samples were incubated with either 2 µg CREB1 antibody (Abcam: ab31387), 2 µg of phospho-CREB1 antibody (Cell Signaling: 9198), 2 µg of p53 antibody (1C12, Cell Signaling 2524), 2 µg of CBP (C-1, Santa Cruz: sc-7300) and 2 µg of control rabbit IgG (Merck Millipore), or no antibody overnight at 4 °C with rotation. Complexes were collected for 1 h at 4 °C with rotation with 60 µl of protein A sepharose beads (Invitrogen) that had been pre-equilibrated in 1:10 Non-Ionic lysis buffer for 1 h. Beads were washed one time each with Low Salt buffer (0.1% SDS, 1% Triton X-100, 2 mM EDTA, 20 mM Tris-HCl pH8.1, 150 mM NaCl), High Salt buffer (0.1% SDS, 1% Triton X-100, 2 mM EDTA, 20 mM Tris-HCl pH8.1, 500 mM NaCl) and LiCl buffer (0.25 M LiCl, 1% NP40, 1% deoxycholate, 1 mM EDTA, 10 mM Tris-HCl pH8.1), followed by two washes with TE buffer. Protein-Protein complexes were eluted from the beads adding 30 μL of 4X Laemilli buffer. The samples were ready for western blot analysis.

### CREB-CBP inhibitor treatment

*p53*^*WT/WT*^ and *p53*^*KO/KO*^ cells and the corresponding OS cells derived from cell lines made from primary tumors were plated at 2500 cells per well in 96-well plates. After 24 h, the cells were treated with 666–15 (Tocris Bioscience) in an 11-point dose response ranging from 0.01 to 10 mmol/L. All assays were performed in duplicate. At 48 and 72 h after treatment, the cell viability for each well was quantified on the basis of the direct measurement of intracellular ATP using the ATP-Lite luminescent assay (PerkinElmer). All luminescent measurements were recorded on the EnSpire plate reader (PerkinElmer). Data were plotted and the IC_50_ value calculated using Prism 6 software.

### Bioinformatics and data mining and selection of target genes

Creb1 target genes were selected from^38–40^. We only validated genes which were considered related to cAMP signaling. For validation of p53 direct targets we referred to^41^ that describes a genome-wide approach to detect lnc-RNAs and p53 transcriptional targets in human cells. We downloaded the ChIP-seq data for p53 in MEFs from^42^. We therefore compared the human p53 ChIP-seq data with previously published p53 ChIP-seq data from mouse embryonic fibroblasts (MEFs) treated with the DNA-damage drug doxorubicin. A subset of genes which were doxorubicin induced and were common in human and mouse datasets were selected.

### Statistical analysis

Data were presented as mean ± SEM. Statistical comparisons were performed in Prism 6.0 unless otherwise indicated. Parametric Student’s *t*-test, area under the curve or 2-way ANOVA with multiple comparison test were used for comparisons with *P* < 0.05 considered as significant.

## Results

### Acute loss of p53 in primary osteoblasts activates PTHrP-cAMP-Creb1 signalling

**Fig. 1:**
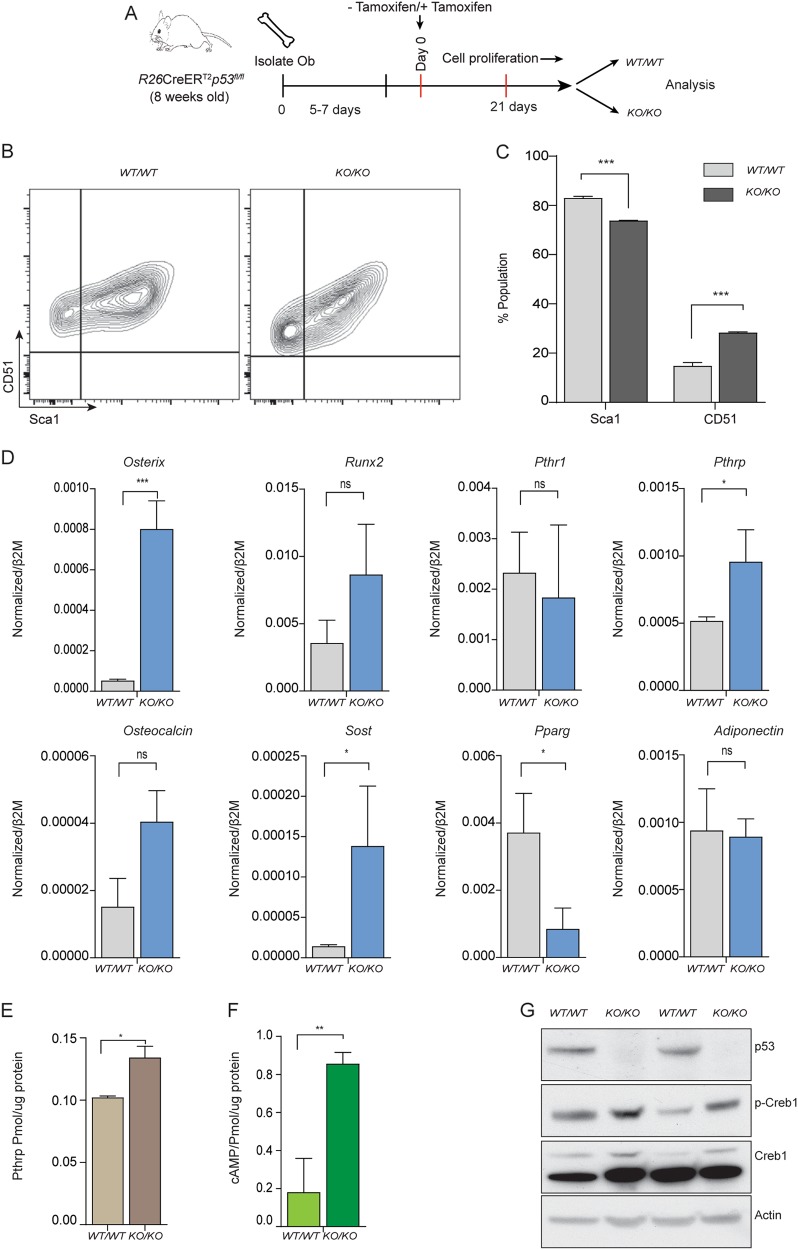
Loss of p53 in primary osteoblasts leads to accelerated differentiation and increased PTHrP-cAMP-Creb1 axis. **a** Schematic representation of generation of independent *p53*^*WT/WT*^ and *p53*^*KO/KO*^ cells. **b** *p53*^*WT/WT*^ and *p53*^*KO/KO*^ cells assessed for expression of Sca-1, CD51, representative results shown, *n* = 3 independent experiments. **c** Cell surface markers from each cell line (*n* = 3 independent cultures). **d** mRNA for *Osterix*, *Runx2*, *Pthr1*, *Pthlh*, *Osteocalcin*, *Sost*, *Pparg*, *Adiponectin* by qPCR in p53 WT and p53 KO cells (normalized to *β2m*; mean ± SEM, *n* = 3). **e** PTHrP in lysates of *p53*^*WT/WT*^ and *p53*^*KO/KO*^; pmol/µg of protein using radioimmunoassay. Data pooled from 3 independent cultures; mean ± SEM. **f** cAMP accumulation (pmol/µg of protein) after treatment with 100 μM IBMX for 30 mins in *p53*^*WT/WT*^ and *p53*^*KO/KO*^. Data pooled from 3 independent cultures; mean ± SEM. **g** Western blot of p53, pCREB1, and Creb1, *β*-Actin used as a loading control. Data representative of 2 independent cell lines from each. See Figure S1. For all panels: *P < 0.05, **P < 0.01, ***P < 0.001

We modelled the acute loss of p53, in isolation of other genetic changes, using primary osteoblastic cells isolated from long bones of *R26*-CreER^T2ki/+^*Tp53*^*fl/fl*^ (referred to as KO herein) and *R26*-CreER^T2ki/+^*Tp53*^*+/+*^ (referred to as WT) animals. Cells were isolated and expanded for 5–7 days. The cells were then cultured in proliferative media and treated + /− tamoxifen to generate isogenic cultures. Tamoxifen treatment was continued until day 21 when it was stopped. The cells were genotyped to confirm they were wild-type or *Tp53*-deficient then used for the assays described. For all subsequent experiments, excluding those describing results from differentiation cultures, the cells were plated and allowed to reach ~70–80% confluence then used for experiments (for example, treated with IBMX or forskolin). For differentiation experiments, the cells were plated at the appropriate cell density (no tamoxifen added) and placed in differentiation inducing media for a further 21 days. Isogenic cell pairs (*Tp53* WT and *Tp53* KO) were made in order to allow comparison of cells from the same animal (Fig. 1a).

Analysis of proliferative cultures immediately after cessation of tamoxifen treatment demonstrated that the expression of p53 target genes was diminished in the KO cultures, compared to non-tamoxifen treated isogenic cultures^8^. By cell surface phenotype, expression of CD51 was increased and Sca1 was decreased on KO cells compared to WT counterparts (Fig. 1b, c). At the completion of tamoxifen treatment, when deletion of p53 was confirmed, there was increased expression of *Osterix*, *Pthrp*, and *Sost* in KO cells, with no significant increase in other osteoblast lineage markers *Pthr1* and *Runx2* (Fig. 1d). A clearer indication of the effect of *Tp53* deficiency was obtained by examining differentiation potential of the WT and *Tp53* deleted cells by culturing for a further 21 days in osteogenic differentiation media. Expression of osteoblastic lineage markers remained stable in the KO cells and generally at a higher level than in the p53 WT cells during differentiation (Supplemental Fig. 1A, B). Adipogenic markers *Pparg* and *Adiponectin* changed comparably between genotypes (Supplemental Fig. 1A, B). These findings are consistent with previous reports that p53 loss is associated with altered osteoblast differentiation^32,43^.

We assessed the effect of *Tp53* deficiency on PTHrP-cAMP-Creb1 pathway status. KO cells had increased PTHrP protein levels and intracellular cAMP accumulation compared to p53 WT counterparts (Fig. 1e, f). Consistent with these findings, there were higher levels of basal phospho-Creb1 in p53-deficient cells (Fig. 1g). Examination of the kinetics of p53 protein expression during differentiation revealed that in WT cells, p53 protein level gradually reduced, concurrent with a reduction of Creb1 (Supplemental Fig. 1C, D). However, p53-deficient osteoblasts retained Creb1 expression over the differentiation time course, including phosphorylated active Creb1 (Supplemental Fig. 1C, D). This result is consistent with the persistence of Creb1 and phospho-Creb1 in OS cells induced to differentiate in vitro^8^.

### Elevated cAMP induces apoptosis that is regulated by p53

**Fig. 2:**
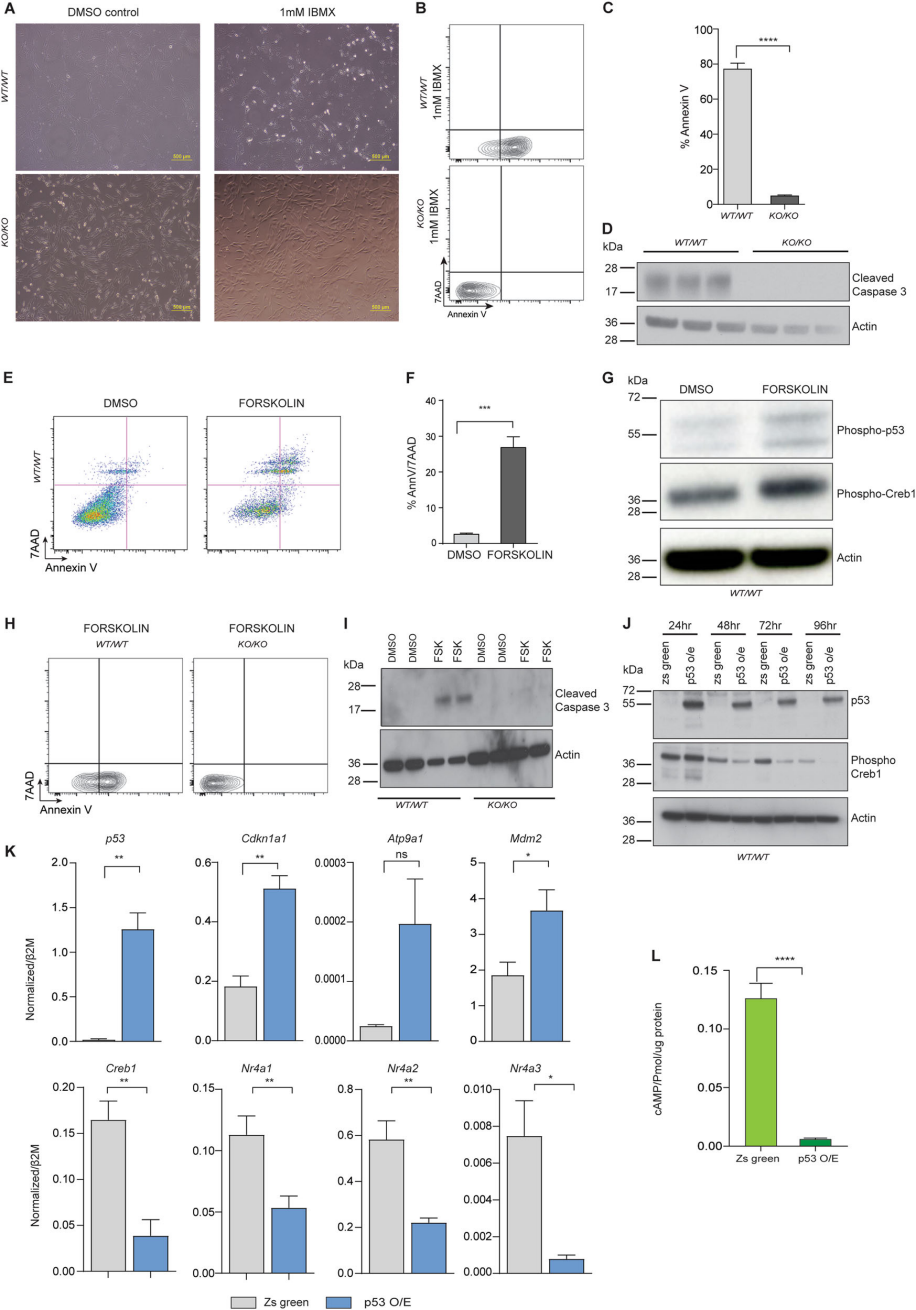
cAMP-induced apoptosis in normal osteoblast is negatively regulated by p53. **a** *p53*^*WT/WT*^ and *p53*^*KO/KO*^ cells treated with 1 mM IBMX; representative image of 3 independent isogenic cells. **b** AnnexinV/7-AAD profiles of IBMX-treated *p53*^*WT/WT*^ and *p53*^*KO/KO*^ . **c** Percent apoptotic cells in each culture *p53*^*WT/WT*^ and *p53*^*KO/KO*^ post-IBMX treatment (mean ± SEM, *n* = 3). **d** Apoptosis shown by cleaved caspase-3 activation in 3 independent *p53*^*WT/WT*^ and *p53*^*KO/KO*^ isogenic cells. **e** Annexin V and 7AAD profiles of forskolin-treated *p53*^*WT/WT*^ primary osteoblasts cells. **f** Percent apoptotic cells in each culture *p53*^*WT/WT*^ pre- and post-forskolin treatment (mean ± SEM, *n* = 3). **g** Western blot of phospho-p53, phospho-Creb1 with *β*-Actin as a loading control. Data representative of 2 independent cell lines from each. **h** AnnexinV and 7AAD profiles of forskolin-treated *p53*^*WT/WT*^ and *p53*^*KO/KO*^ primary osteoblasts cells. **i** Presence of cleaved caspase-3 in 3 independent *p53*^*WT/WT*^ and *p53*^*KO/KO*^ isogenic cells. **j** Western blot of p53, phospho-Creb1 with *β*-Actin as a loading control. Data representative of 2 independent cell lines from each. **k** mRNA for *p53*, *Cdkn1a1*, *Atp9a1*, *Mdm2*, *Creb1*, *Nr4a1*, *Nr4a2*, *Nr4a3* by qPCR in p53^*WT/W*^ cells at 72 h after overexpression of p53. Expression levels normalized to *β2m*; mean ± SEM (*n* = 3). **l** Intracellular cAMP accumulation (pmol/µg protein; cells treated with 100 μM IBMX for 30 mins) in *p53*^*WT/WT*^ cells overexpressed with p53; data pooled from 3 independent cultures; mean ± SEM. For all panels: *P < 0.05, **P < 0.01, ***P < 0.001

As intracellular cAMP accumulation increased after p53 deletion from primary osteoblasts (Fig. 1f), we sought to determine the effect of acutely elevating cAMP levels on wild type osteoblastic cells. The cells were treated with DMSO (control) or IBMX (1 mM), which inhibits the phosphodiesterases (PDE) that degrade cAMP, to enable accumulation of the cells’ intrinsic cAMP. Within 1 h of IBMX treatment there was a major difference in cell morphology, with WT cells poorly attached to the culture dish compared to the KO (Fig. 2a). There was a significant increase in AnnexinV positive cells in the WT culture (Fig. 2b, c). Cell death/apoptosis was assessed by measuring the levels of cleaved caspase-3^44^. Upon preventing breakdown of intracellular cAMP, cleavage of caspase-3 was rapidly induced in WT cells unlike the KO counterpart (Fig. 2d).

As an alternative approach to persistently elevate cAMP, cells were treated with the direct cAMP stimulus forskolin (Supplemental Figure 2A). After 24 h of forskolin treatment, p53 WT cells had an altered morphology similar to the IBMX-treated cells (data not shown). WT cells underwent cell death within 24 -hours of forskolin treatment as quantitated by AnnexinV/7AAD staining (Fig. 2e, f). Elevation of cAMP via forskolin resulted in increased phosphorylation of Creb1, as expected, and also of p53 (Fig. 2g). p53 KO cells were protected from cell death induced by forskolin treatment (Fig. 2h, i). This occurred despite the p53 KO cells generating higher intracellular cAMP levels from the same dose of forskolin than the WT cells (Supplemental Figure 2A).

The deletion of p53 alone resulted in an increase in intracellular cAMP and phosphorylation of Creb1 (Fig. 1f, g). We tested if overexpression of p53 could suppress the cAMP-Creb1 axis. Primary osteoblasts were infected with lentivirus overexpressing murine p53 (Fig. 2j, k). Downstream transcriptional targets of p53 were significantly increased in expression (Fig. 2k) and, strikingly, Creb1 targets were repressed (Fig. 2k). This result was confirmed independently in HEK293T cells overexpressing human P53 (Supplemental Fig. 2C, D). The overexpression of p53 also led to a profound reduction in intracellular cAMP formation in the cells (Fig. 2l), and a reduction of Creb1 phosphorylation (Fig. 2j). Thus, overexpression of p53 repressed cAMP accumulation and reduced Creb1 activity.

### Accumulation of cAMP in p53-deficient cells activates proliferative networks

**Fig. 3:**
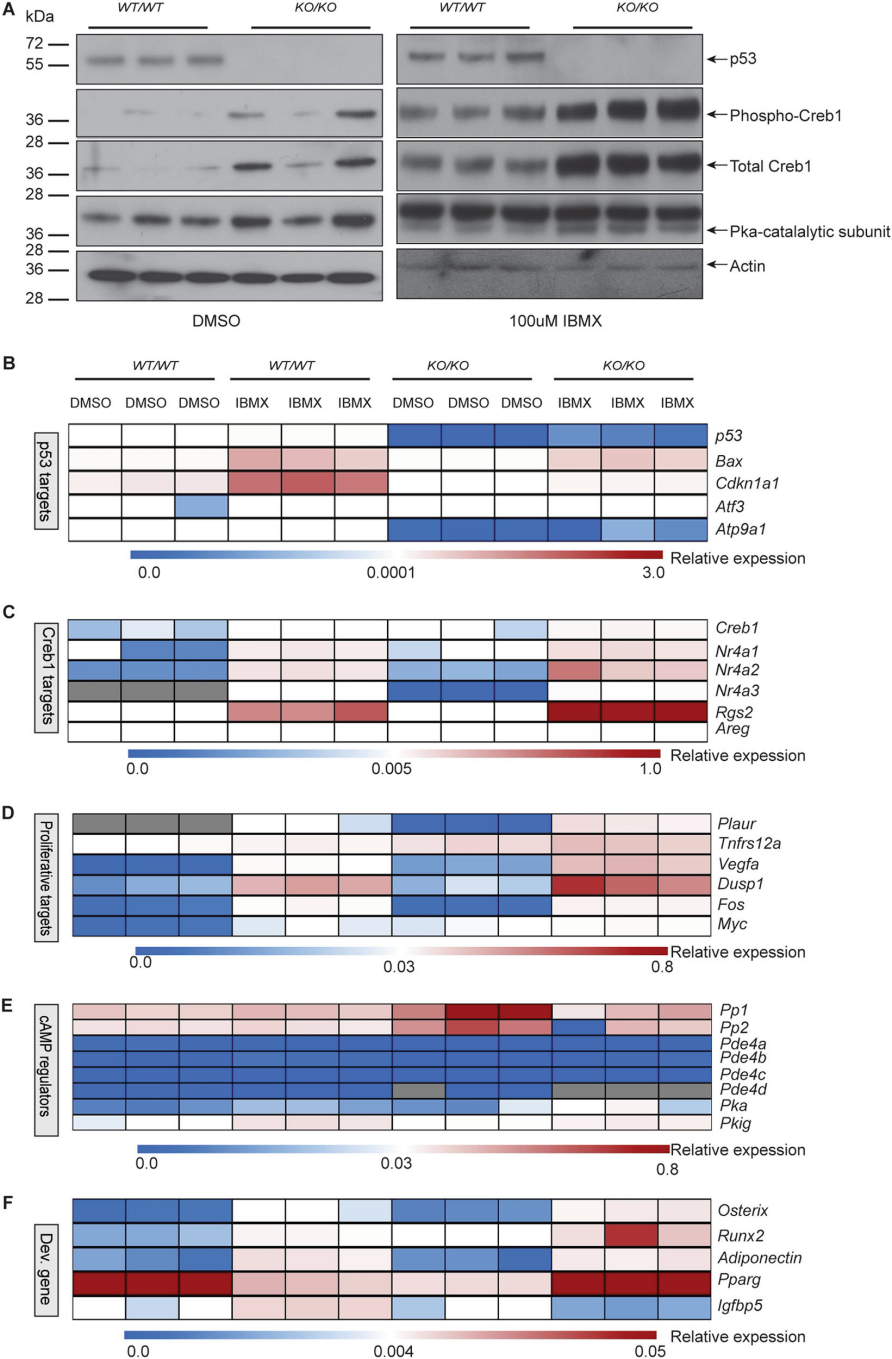
Accumulation of cAMP in *p53*^*KO/KO*^ cells leads to activation of pro-proliferative signatures. **a** Western blot of p53, phospho-Creb1 and Creb1, PKA (catalytic subunit); *β*-Actin as loading control. Data representative of three independent isolates of isogenic *p53*^*WT/WT*^ and *p53*^*KO/KO*^. Cells were treated with 1 mM IBMX as indicated in methods. **b** Heat map of qPCR data. Expression of the p53 target genes between indicated cell types; three independent isogenic cell lines for each, expressed as mRNA normalized to *β2m*. **c** Heat map of qPCR data. Expression of Creb1 target genes between indicated cell types; > 3 independent isogenic cell lines for each, expressed as mRNA normalized to *β2m*. **d** Heat map of qPCR data for genes important in cellular proliferation. Expression of the Creb1 target genes between indicated cell types.; > 3 independent isogenic cell lines for each, expressed as mRNA normalized to *β2m*. **e** Heat map of qPCR data for genes that are negative regulators of cAMP. Expression of the genes between indicated cell types; three independent isogenic cell lines for each, expressed as mRNA normalized to *β2m*. **f** Heat map of qPCR data for genes important in development. Expression of the genes between indicated cell types; > 3 independent isogenic cell lines for each, expressed as mRNA normalized to *β2m*. Data pooled from 3 independent cultures; mean ± SEM. See Figure S3. For all panels: *P < 0.05, **P < 0.01, ***P < 0.001

WT cells and isogenic KO counterparts were treated with DMSO (control) or 100 μM IBMX for 1 h and the expression/activation of the downstream cAMP-PKA pathway monitored. Analysis of independently derived cell cultures revealed increased levels of the PKA-catalytic subunit and phospho-Creb1 in KO cells. There was a basal activation of phospho-Creb1 in p53 KO cells as compared to p53 WT cells (Fig. 3a).

To confirm that the cAMP accumulated following IBMX treatment, as seen in Fig. 1f, resulted in activation of signaling qRT-PCRs were performed. The deletion of p53 resulted in an expected decreased expression of *Tp53* and p53 target genes (Fig. 3b, Supplemental Figure 3A) and was associated with activation of Creb1 target genes as we previously described^7,8^ (Fig. 3c, Supplemental Fig. 3B). Loss of p53 was associated with an increased expression of *Ras* and *Myc*^45,46^. To address this finding, we selected several targets known to be regulated by *Myc* and *Ras*^47,48^. Transcriptional target genes of both Myc and Ras were significantly upregulated in p53 KO cells (Fig. 3d, Supplemental Figure 3C). In human OS, mutations in components of the cAMP pathway have been defined, including in the PDE family, A kinase anchoring proteins (AKAP) and protein phosphatases (PP)^1^. We tested whether loss of p53 directly affected the expression of these genes in WT and KO osteoblasts. The majority of genes were not different between the genotypes, apart from a complete loss of *Pde4d* mRNA in p53 KO cells (Fig. 3e, Supplemental Fig. 3D). Pde4d hydrolyzes cAMP and results in its breakdown^49^. Expression of markers of osteoblast differentiation were upregulated in p53 KO cells compared to the isogenic counterpart (Fig. 3f, Supplemental Fig. 3E).

### Activation of p53 directly suppresses Creb1

**Fig. 4:**
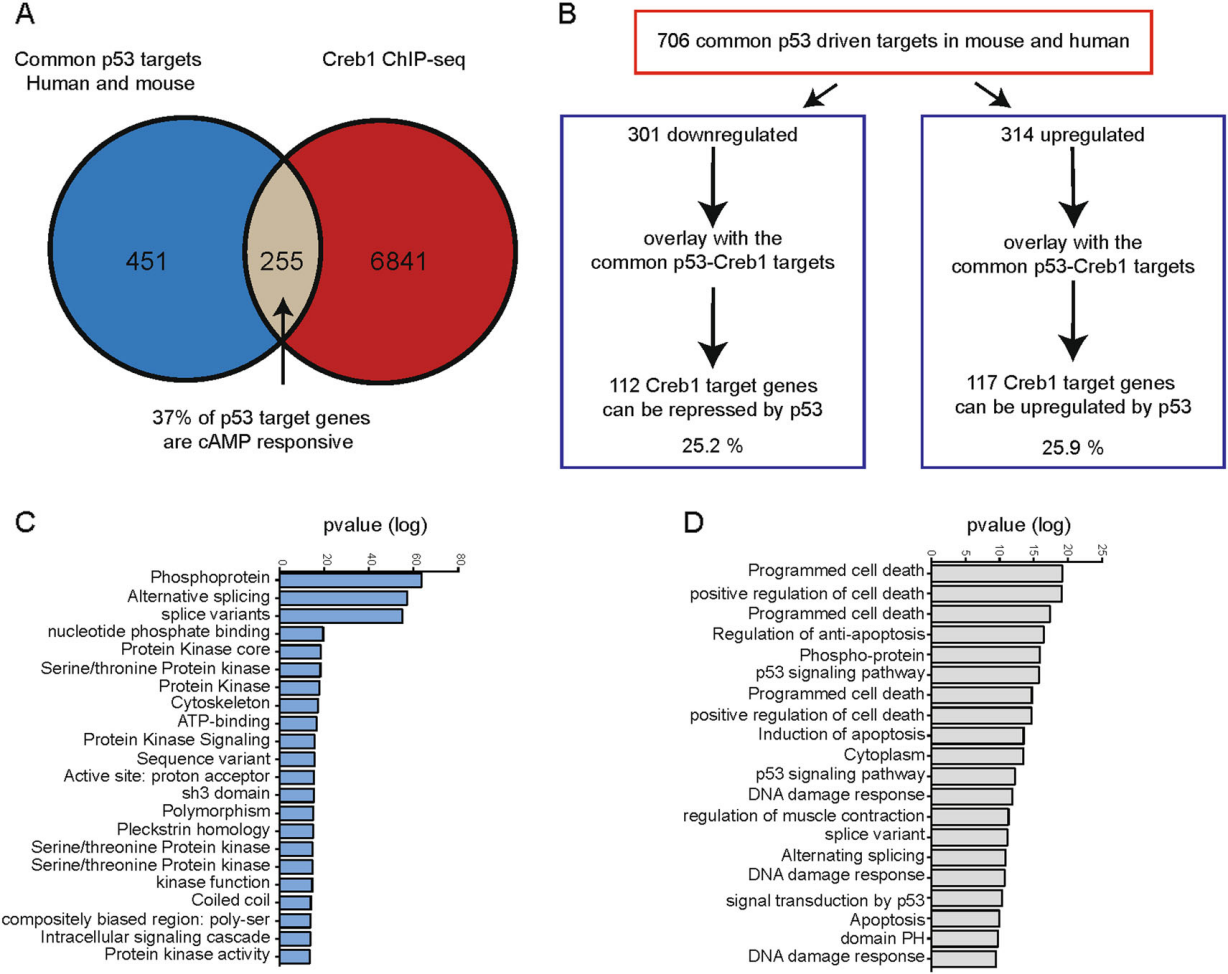
In silico analysis of p53 and Creb chromatin targets. **a** Venn diagram depicting the overlap and outliers between common human and mouse p53 targets and Creb1 ChIP-seq dataset. **b** Table showing the bifurcation of up- and downregulated Creb1 targets regulated by p53. **c** Pathways governed by p53-mediated downregulated Creb1 targets. **d** Pathways governed by p53-mediated upregulation of Creb1 targets

We next asked if genome-wide gene signatures associated with cAMP-PKA were impacted by loss of p53 in silico. Creb1 and P53 target genes were identified from ChIP-seq studies^39,41^. The P53 target genes were further refined against a second independent dataset of p53 ChIP-seq from murine embryonic fibroblasts^42^. When overlapped, ~37% of Creb1 targets could also be directly regulated by p53 (Fig. 4a). Of the common p53-Creb1 targets, the genes were then separated into groups that were either up (*n* = 314) or down (*n* = 301) regulated by p53, respectively, and pathway analysis performed using DAVID^50^ (Fig. 4b). The functions of the genes repressed by p53 were over-represented in pathways related to protein kinase signaling (Fig. 4c), whereas the genes that could be upregulated by p53 were associated with classical p53 signatures including cell cycle arrest and apoptosis (Fig. 4d).

**Fig. 5:**
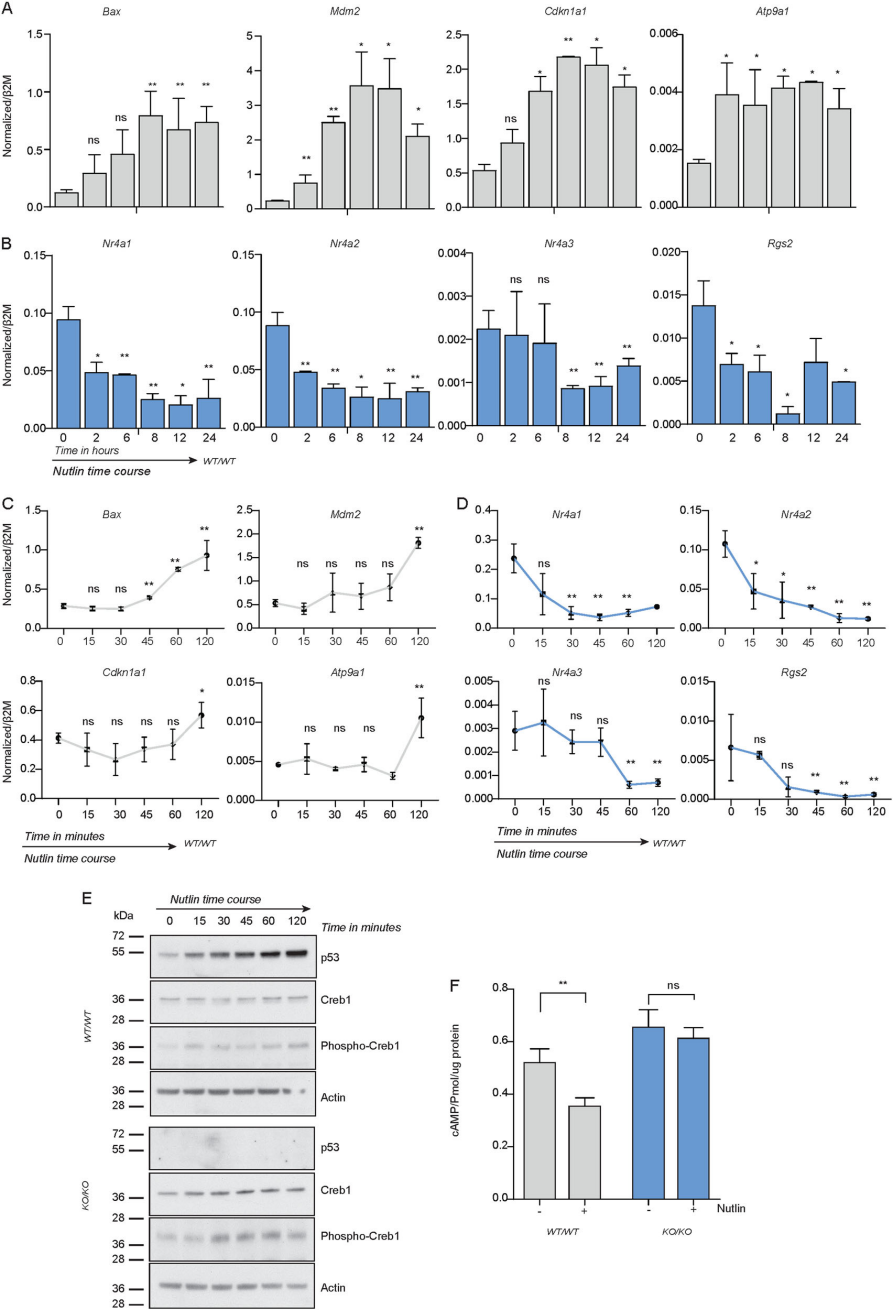
Activation of p53 directly suppresses Creb1 function. **a** qPCR validation of p53 target gene expression following 10 μM Nutlin treatment over time course of 24 h; 3 independent isogenic *p53*^*WT/WT*^ cells, mean ± SEM. **b** qPCR validation of Creb1 target gene expression following 10 μM Nutlin treatment over time course of 24 h; 3 independent isogenic *p53*^*WT/WT*^ cells, mean ± SEM. **c** qPCR validation of p53 target gene expression following 10 μM Nutlin treatment over time course of 2 h; 3 independent isogenic *p53*^*WT/WT*^ cells, mean ± SEM. **d** qPCR validation of Creb1 target gene expression following 10 μM Nutlin treatment over time course of 2 h; 3 independent isogenic *p53*^*WT/WT*^ cells, mean ± SEM. **e** Western blot of p53, Phospho-Creb1 and Creb1 post-Nutlin-3a treatment for time course of 2 h; *β*-Actin used as a loading control; Data representative of 2 independent isogenic cell lines. **f** Intracellular cAMP formation (pmol/μg protein) in *p53*^*WT/WT*^ and *p53*^*KO/KO*^ (IBMX 100 μM) 30 mins post-Nutlin treatment; data pooled from 3 independent cultures; mean ± SEM. See figure S4. For all panels: *P < 0.05, **P < 0.01, ***P < 0.001

To directly test the link between p53 and protein kinase signaling we used a pharmacogenetic approach to examine how these pathways interacted when activated individually or concurrently. WT osteoblasts were treated with the MDM2 inhibitor Nutlin-3a to activate WT p53 in a time course of 24 h. qRT-PCR demonstrated a significant activation of p53 target genes in a temporal fashion (Fig. 5a). There was a reciprocal relationship between p53 activation and the expression of Creb1 target genes (Fig. 5b). The downregulation of Creb1 activity was an early consequence of p53 activation, as Creb1 targets were significantly repressed with 2 h of Nutlin-3a treatment (Fig. 5c, d). Similar inhibition of Creb1 activity occurred when p53 was activated using doxorubicin, a genotoxic drug used to treat a number of cancer including OS (Supplemental Fig. 4A–C). Western blot analysis confirmed the increase in p53 by Nutlin-3a treatment in p53 WT cells (Fig. 5e). In assessing this further, Nutlin-3a mediated activation of p53 significantly decreased intracellular cAMP levels in WT osteoblasts and this effect was completely prevented in KO cells (Fig. 5f). The activation of p53 therefore leads to reduced formation of intracellular cAMP and a repression of Creb1 transcriptional activity.

### Simultaneous p53 activation and elevated cAMP levels induce apoptosis

**Fig. 6:**
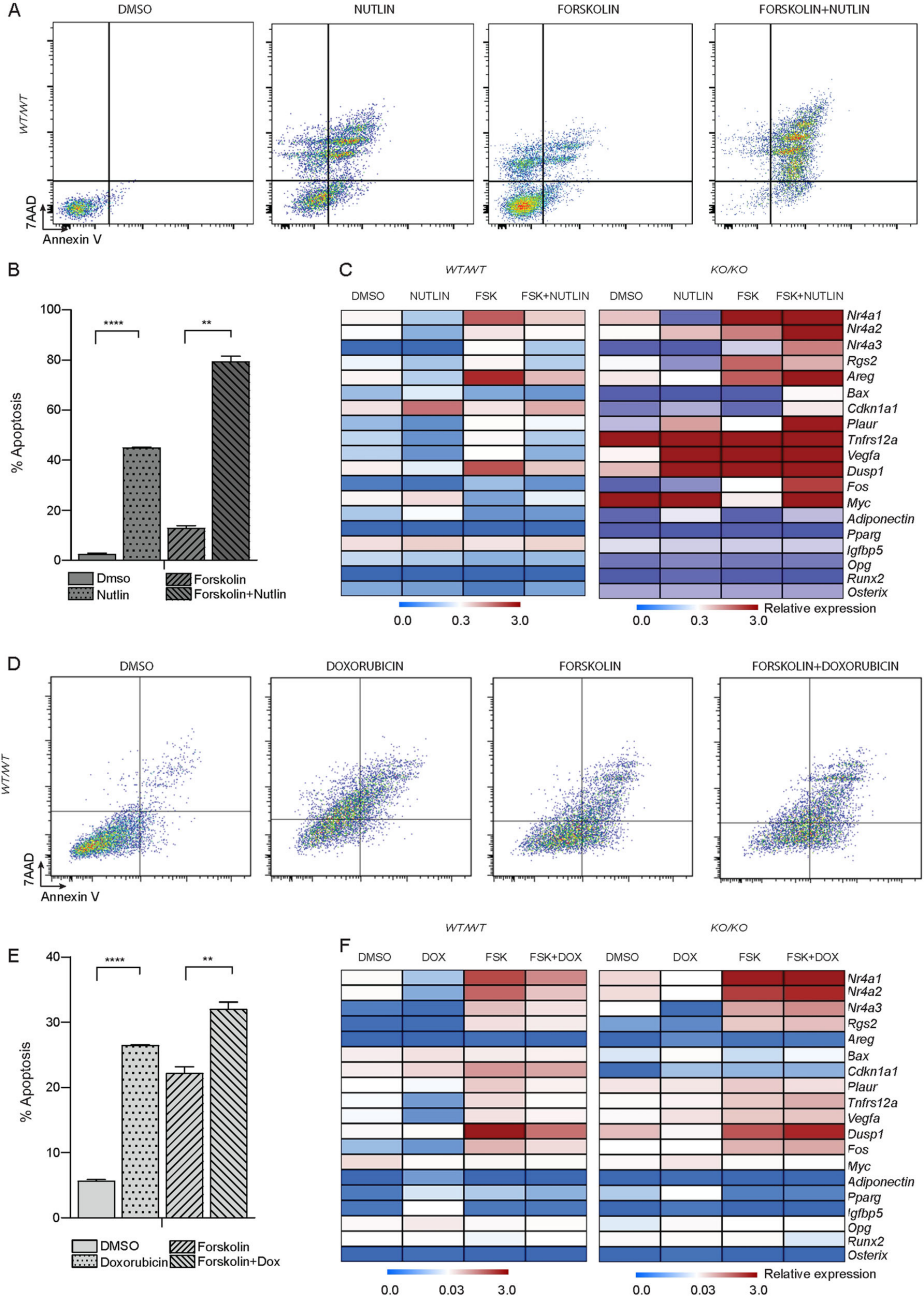
Concurrent activation of p53 and cAMP pathway leads to apoptosis. **a** AnnexinV/7-AAD profiles of *p53*^*WT/WT*^ cells treated with less than 0.1% DMSO, 10 μM Nutlin, 10 μM Forskolin, and combined treatment with Forskolin and Nutlin. **b** Percent apoptotic cells in each treatment group; mean ± SEM. **c** Heat map of qPCR data. Expression of the p53 and Creb1/pro-proliferative targets, developmental targets between cell types; Data from 3 independent isogenic cell lines for each, expressed as relative expression normalized to *β2m*. Data expressed as mean ± SEM (*n* = 3). **d** AnnexinV/7-AAD profiles of *p53*^*WT/WT*^ cells treated with less than 0.1% DMSO, 500 nM Doxorubicin, 10 μM Forskolin and combinatorial treatment of Forskolin and Doxorubicin. **e** Percent apoptotic cells in each treatment group; 3 independent cell lines. **f** Heat map of qPCR data. Expression of the p53 and Creb1/pro-proliferative targets, developmental targets between cell types; Data from 3 independent isogenic cell lines for each, expressed as relative expression normalized to *β2m*. Data expressed as mean ± SEM (*n* = 3). For all panels: *P < 0.05, **P < 0.01, ***P < 0.001. See figure S5. For all panels: *P < 0.05, **P < 0.01, ***P < 0.001

In order to determine whether elevated intracellular cAMP can directly affect the cells’ response to p53 activation, we treated WT and KO osteoblasts with Nutlin-3a alone (p53 activator), forskolin alone (cAMP stimulus) or the combination of both for 24 h and evaluated the cellular effects and gene expression. Elevating cAMP together with activation of p53 led to apoptosis in p53 WT osteoblasts. qRT-PCR was performed revealing that simultaneous activation of p53 and cAMP resulted in the suppression of Creb1 targets, suggesting that the response by p53 was dominant over the cAMP-induced response (Fig. 6a, b). There was no significant effect on the expression of genes important for osteoblastic lineage development such as *Osterix* and *Runx2* (Supplemental Fig. 5A). This effect was rapid, seen within 1 h. The transcriptional effect of p53 activation and Creb1 signature suppression was absent in p53 KO cells, which confirmed the specificity of this response to p53 (Fig. 6c). A comparable effect was observed when doxorubicin was combined with forskolin, with the cells undergoing apoptosis and Creb1 target genes being suppressed (Fig. 6d–f, Supplemental Fig. 5B). PI staining was performed to assess the cell cycle status of the cells and there was no significant change in cell cycle parameters (Supplemental Fig. 5C), suggesting that at least in the window of treatment there was no obvious changes in cell cycle parameters. This indicates that the ability to tolerate elevated and aberrant cAMP pathway activation in osteoblasts, as is seen in OS, is dictated by p53 status.

### Creb1, Cbp, and p53 can occur in complex

**Fig. 7:**
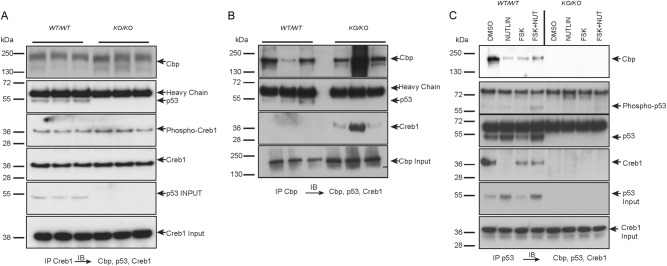
Creb1, Cbp, and p53 interact. **a** Western blot following immunoprecipitation of Creb1 and immunoblot with Cbp, p53 and Creb1 from 3 independent isogenic *p53*^*WT/WT*^ and *p53*^*KO/KO*^. **b** Western blot following Cbp immunoprecipitation and immunoblot with Cbp, p53, and Creb1 from 3 independent isogenic *p53*^*WT/WT*^ and *p53*^*KO/KO*^. **c** Western blot analysis of p53 immunoprecipitation and immunoblot with Cbp, p53, Creb1, and phospho-Creb1 from isogenic *p53*^*WT/WT*^ and *p53*^*KO/KO*^ cells treated with DMSO, Nutlin, Forskolin, and Forskolin + Nutlin

Phosphorylation of CREB mediates recruitment of co-activator associated complex of Creb1 (CBP) to p53-responsive promoters, through potentially a direct interaction with p53^36^. In whole cell lysates from p53 WT and KO cells, steady state Creb1 and phospho-Creb1 were significantly higher (Figs. 1, 2). To assess if there was a direct interaction between p53 and Creb1, Creb1 was immunoprecipitated and the Creb1-containing complexes from p53 WT cells assessed with p53 KO cells used as a control. In the WT cells, p53 was co-immunoprecipitated with both Creb1 and phospho-Creb1-containing protein complexes (Fig. 7a). We assessed whether Cbp was also associated with Creb1 complexes in these cells. When Creb1 was immunoprecipitated in both cell types, it could be found associated with Cbp (Fig. 7b). Interestingly, when Cbp containing complexes were immunoprecipitated from p53 WT and KO cells, it revealed a different interaction. In the presence of p53, Cbp could interact with p53, but there was greatly reduced interaction with Creb1 (Fig. 7a). However, with the loss of p53 there was enhanced interaction of Creb1 with Cbp (Fig. 7b). Whilst the consequences of this are not immediately clear, they suggest that p53 status may regulate the extent of interaction of Creb1 and Cbp.

We further sought to understand how the dynamics of the p53-Creb1-Cbp interactions changed in response to activation of p53 or cAMP. WT and KO cells were treated with DMSO, Nutlin-3a, forskolin, or forskolin/Nutlin-3a followed by immunoprecipitation with p53. Following p53 activation there was a decreased amount of Creb1 associated with p53 (Fig. 7c). When cAMP levels were increased in isolation of p53 activation, there was evidence of the p53-Creb1-Cbp interaction, however there was less p53 associated with this complex. When both p53 and cAMP pathways were activated concurrently, the strongest association between these proteins could be seen.

### Inhibition of the Creb1-Cbp interaction as a potential therapeutic strategy for osteosarcoma

**Fig. 8:**
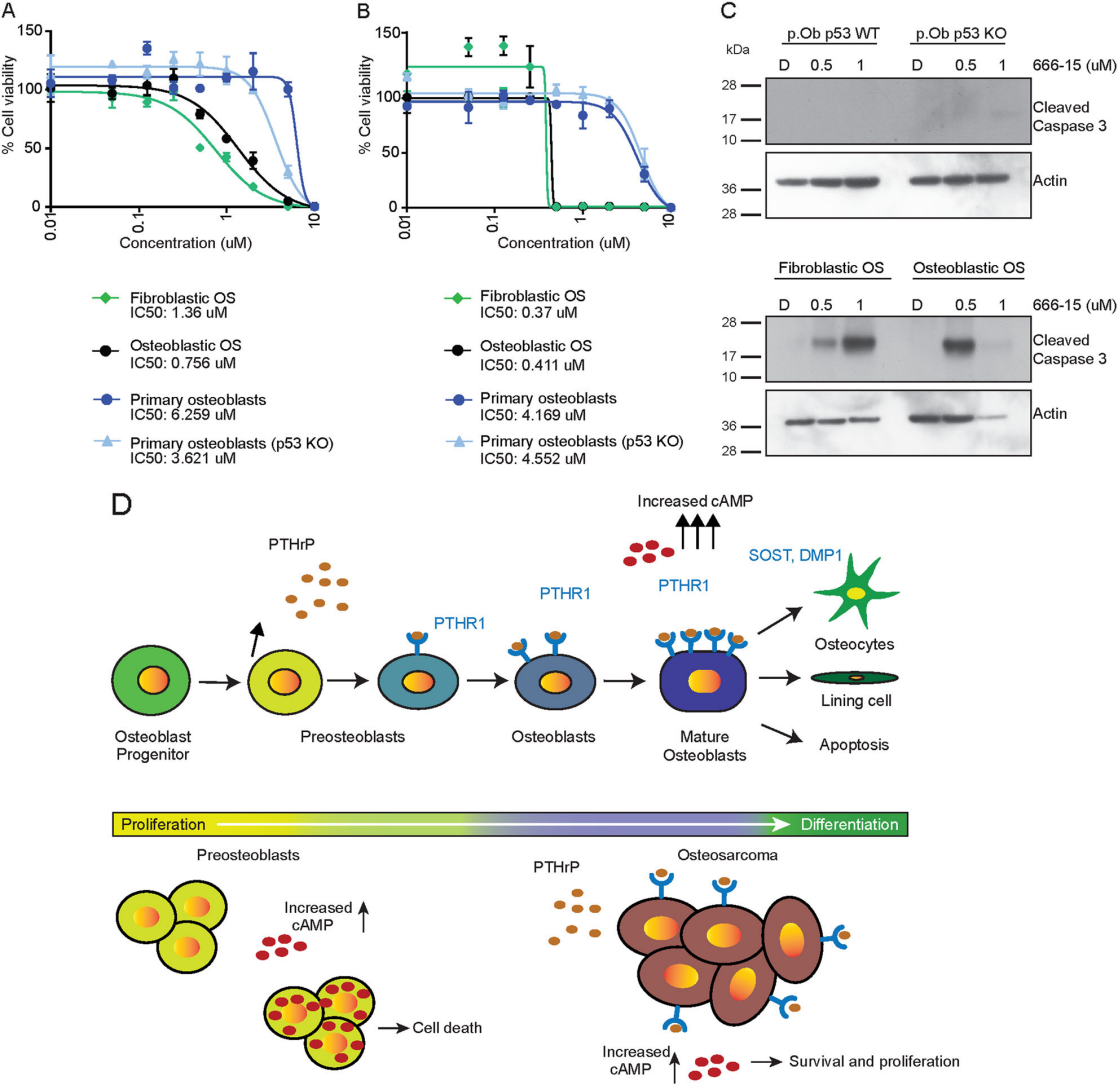
Inhibiting the interaction of Creb1-Cbp leads to toxicity in p53 null osteosarcoma cells. **a** Dose response curves of cells treated with 666–15 Creb1-Cbp inhibitor for 48 h. **b** Dose response curves of cells treated with 666–15 Creb1-Cbp inhibitor for 72 h. **c** Western blot analysis for cleaved caspase-3 from the indicated cells treated for 48 h with the Creb1-Cbp interaction inhibitor 666–15. **d** Graphical summary of the differences between lineages committed pre-osteoblasts and the sequence of expression of paracrine action of PTHrP on differentiating cells expressing PTHR1. Pre-osteoblasts cannot tolerate increased cAMP levels as compared to p53-deficient osteosarcoma cells

Several new chemical inhibitors of the CREB pathway have been described, including the CREB inhibitor 666–15^51^. We tested 666–15 against normal osteoblasts (*p53*^*+/+*^), primary p53 KO osteoblasts and primary OS cell cultures isolated from both fibroblastic and osteoblastic OS mouse models^16,22^. A dose response assay demonstrated genotype dependent toxicity after 48 h, which was specific for p53 KO osteoblastic cells and the p53 null OS (Fig. 8a). Normal osteoblasts tolerated high doses of 666–15, unlike OS cells and p53-deficient osteoblasts. Incubating the cells for 72 h with 666–15 led to a more pronounced effect on the OS cells (Fig. 8b). We therefore determined how the cells were dying by measuring the levels of cleaved caspase-3. Inhibition of Creb1-Cbp interaction led to apoptosis in the OS cells but not in normal osteoblasts (Fig. 8c). This result suggested that the Creb1-Cbp interaction is required for the maintenance of proliferation of p53-deficient osteoblasts and, more importantly, the viability of OS cells.

## Discussion

P53 is a negative regulator of key cell fate-mediated programs which, when perturbed, can lead to tumor formation^7,16,20–22,52^. OS, unlike most tumor types, frequently harbors null alleles of *TRP53*^1,3^. The reason for this mutational preference in osteoblastic cells, the lineage of origin of OS^22^, is not clear. Evidence for elevated and persistent signalling through the cAMP/PKA axis in OS has come from mouse genetic models, including those we have developed^6–9^, as well as from mutational analysis of human OS^1^, all leading to the view that deregulation of this pathway plays a critical role in OS^6–8^. In the present work, we have studied the nexus between p53 and cAMP pathways in normal osteoblastic cells. Persistently elevated cAMP levels induce cellular stress in osteoblastic cells, and p53 counteracts this by inducing apoptosis (Fig. 8d). In the absence of p53, a state sufficient to initiate OS, osteoblastic cells become tolerant to elevated and otherwise toxic levels of cAMP/Creb1 pathway activity (Fig. 8d).

We have utilized a reductionist cellular model to understand the consequences of the key mutation in OS, p53 loss, in primary osteoblasts. In this model there was a coordinated increase in cAMP and activated Creb1 levels and transcriptional activity as osteoblastic cells became p53-deficient, prior to transformation into OS. In addition, there was increased expression of *Pthlh* and PTHrP, which we have shown previously to be a critical autocrine stimulus of the cAMP-Creb1 pathway in OS^6–8^. When WT primary osteoblasts were prompted to undergo osteogenic differentiation in culture, p53 and Creb1 expression declined during differentiation. However, in p53 KO primary osteoblasts, the decrease in Creb1 expression during differentiation was delayed. The loss of p53 thus induces an aberrant differentiation program and depresses the cAMP pathway in osteoblasts.

Undifferentiated osteoblastic cells expressed PTHrP but have low to undetectable levels of PTHR1. In normal osteoblast lineage cells, immature osteoblasts make PTHrP but do not sense it, instead it acts in a paracrine manner on more mature osteoblastic lineage cells^10,14^. However, in OS we previously reported that OS cells make PTHrP and have functional PTHR1, with an autocrine axis maintaining elevated and persistent intracellular cAMP levels in OS (Fig. 8d)^6,8^. To investigate the consequence of maximal accumulation of cAMP in osteoblastic cells, we treated cells with either IBMX (prevents breakdown of cAMP) or used forskolin (directly elevates intracellular cAMP). This demonstrated that excessive intracellular cAMP was poorly tolerated by normal osteoblastic cells, resulting in apoptosis. The loss of p53 was sufficient to enable the cells to survive and tolerate the increased cAMP levels. Strikingly, overexpression of p53 in osteoblastic cells was able to reduce intracellular cAMP formation and subsequent activation of the cAMP-Creb1 pathway. We assessed the interaction of these pathways in WT osteoblasts by simultaneously treating cells with forskolin and Nutlin-3a, alone or in combination. p53 activation (Nutlin-3a) is sufficient to repress downstream target gene activation within the cAMP pathway. Our data indicate that osteoblastic cells do not normally tolerate sustained elevations of intracellular cAMP. Loss of p53 imparts an advantage to osteoblasts to tolerate accumulated cAMP levels. Increased expression/activity of the cAMP-Creb pathway is an early event following p53 loss in osteoblasts. Thus, abrogation of p53 activity is necessary before cells can tolerate elevated cAMP pathway activity and utilize it for tumor initiation. Our data also indicate that OS cells tolerate elevated cAMP levels due to the abrogation of the p53-dependent apoptotic response that normal osteoblastic cells incur following activation of this pathway.

Restoration of p53 tumor suppressor pathway function is a promising anti-cancer strategy^53^. However, such approaches are not feasible in p53 null settings such as OS. New treatment options for OS are sorely needed, with the advances in patient outcome over the last 30 years coming from optimizing application of existing therapies rather than the introduction of new agents. The rapid activation of the cAMP pathway following p53 deletion, and its persistence and amplification in OS, may highlight a therapeutic vulnerability in OS cells. We identified that p53 can interact with Creb1 and its co-activator Cbp. Loss of p53 increased the association of Creb1 with Cbp, further enhancing the cellular reprogramming by enabling the elevated cAMP levels to be reflected transcriptionally. Based on these findings, we explored the option of inhibiting the Creb1-Cbp complex interaction. We used an inhibitor, 666–15, which acts by blocking the interaction of CREB1 with CBP^51^. 666–15 or its preceding compounds have demonstrated preclinical efficacy in vivo in leukemia models^54^. Strikingly, inhibition of the Creb1-Cbp interaction had a selective effect on p53 null cells, with a graded response of increasing sensitivity as cells transition from p53-deficient osteoblasts to OS, sparing normal osteoblasts. This result demonstrates that sustained Creb1-Cbp activity is required for survival of OS cells. Collectively our results demonstrate a close relationship between loss of p53 and tolerance to elevated and persistent cAMP/Creb1 signaling in osteoblasts. These pathways are central to the biology of OS and here we find that they are coupled from the earliest events in the initiation of this tumor. Importantly, our results also demonstrate that this pathway may be therapeutic vulnerable in p53-deficient tumors such as OS.

## Electronic supplementary material

Supplemental Figure 1

Supplemental Figure 2

Supplemental Figure 3

Supplemental Figure 4

Supplemental Figure 5

Supplementary figure legends

Supplementary Table
