# Supplementary figures and images for: Tolerance to sustained activation of the cAMP/Creb pathway activity in osteoblastic cells is enabled by loss of p53
Source: Cell Death Dis. 2018 Aug 28;9(9):844. doi: 10.1038/s41419-018-0944-8 (PMC6113249; doi:10.1038/s41419-018-0944-8)

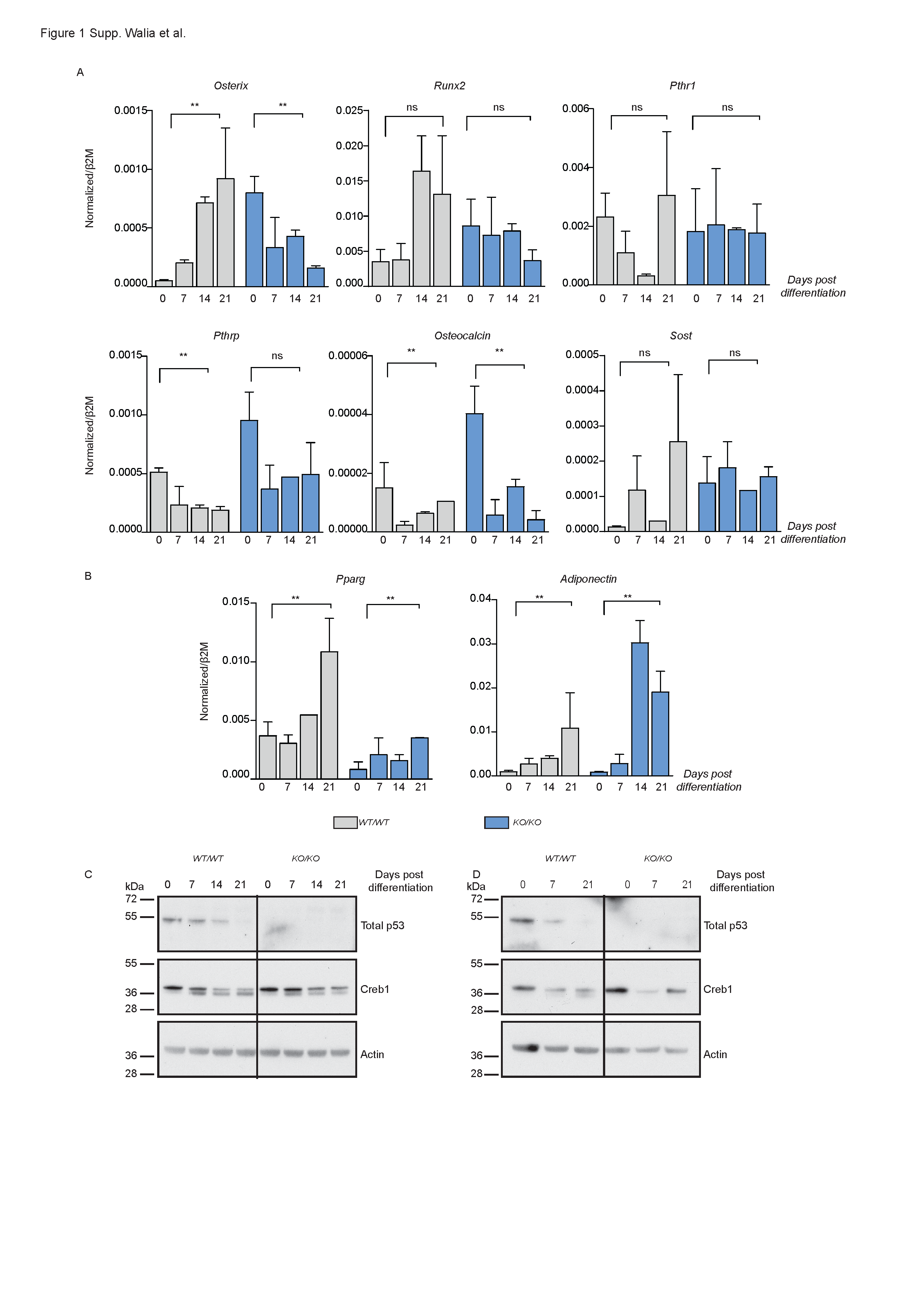

Supplement: Supplementary file 1 — Supplemental Figure 1 [file 41419_2018_944_MOESM1_ESM.tif]

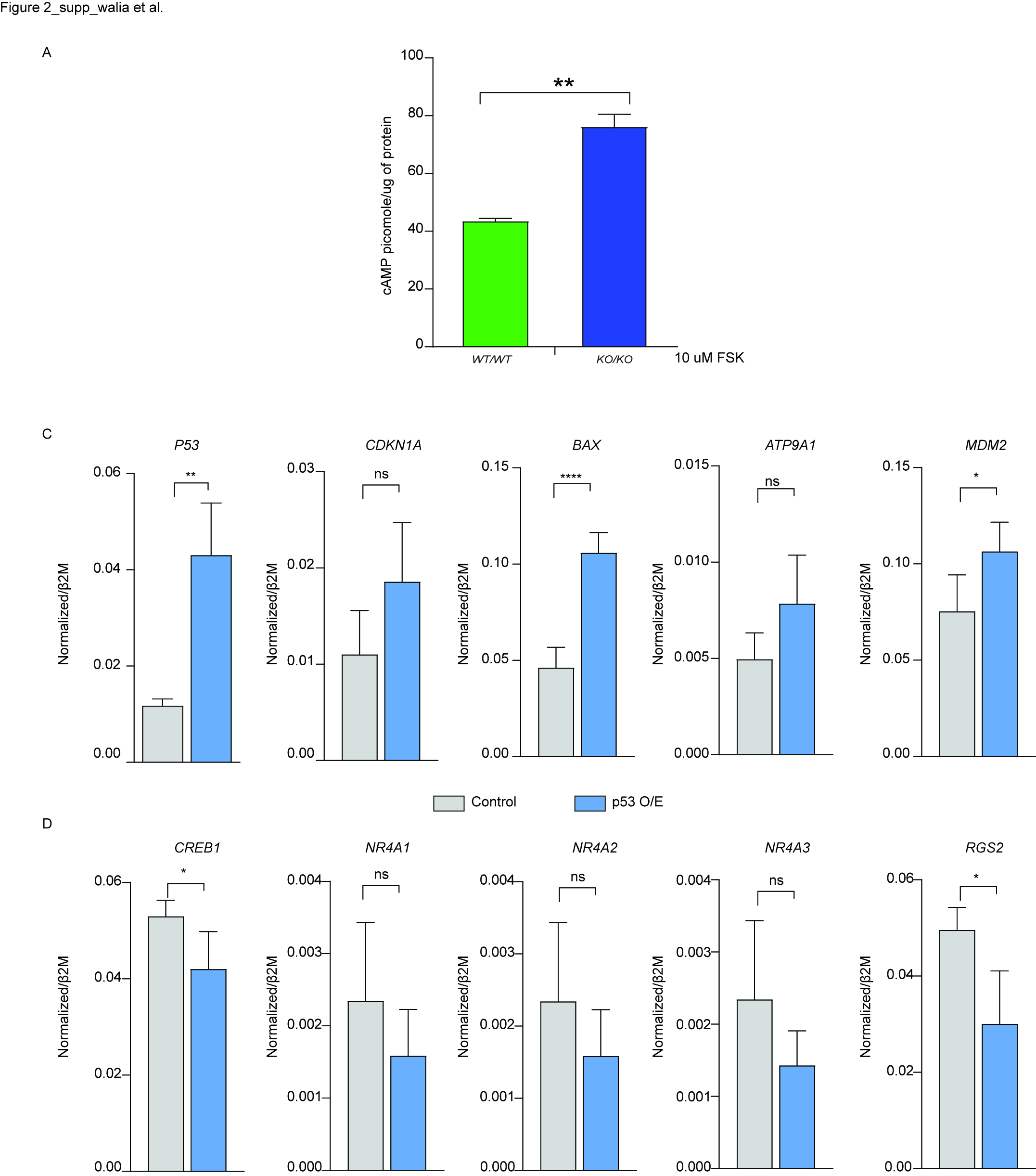

Supplement: Supplementary file 2 — Supplemental Figure 2 [file 41419_2018_944_MOESM2_ESM.tif]

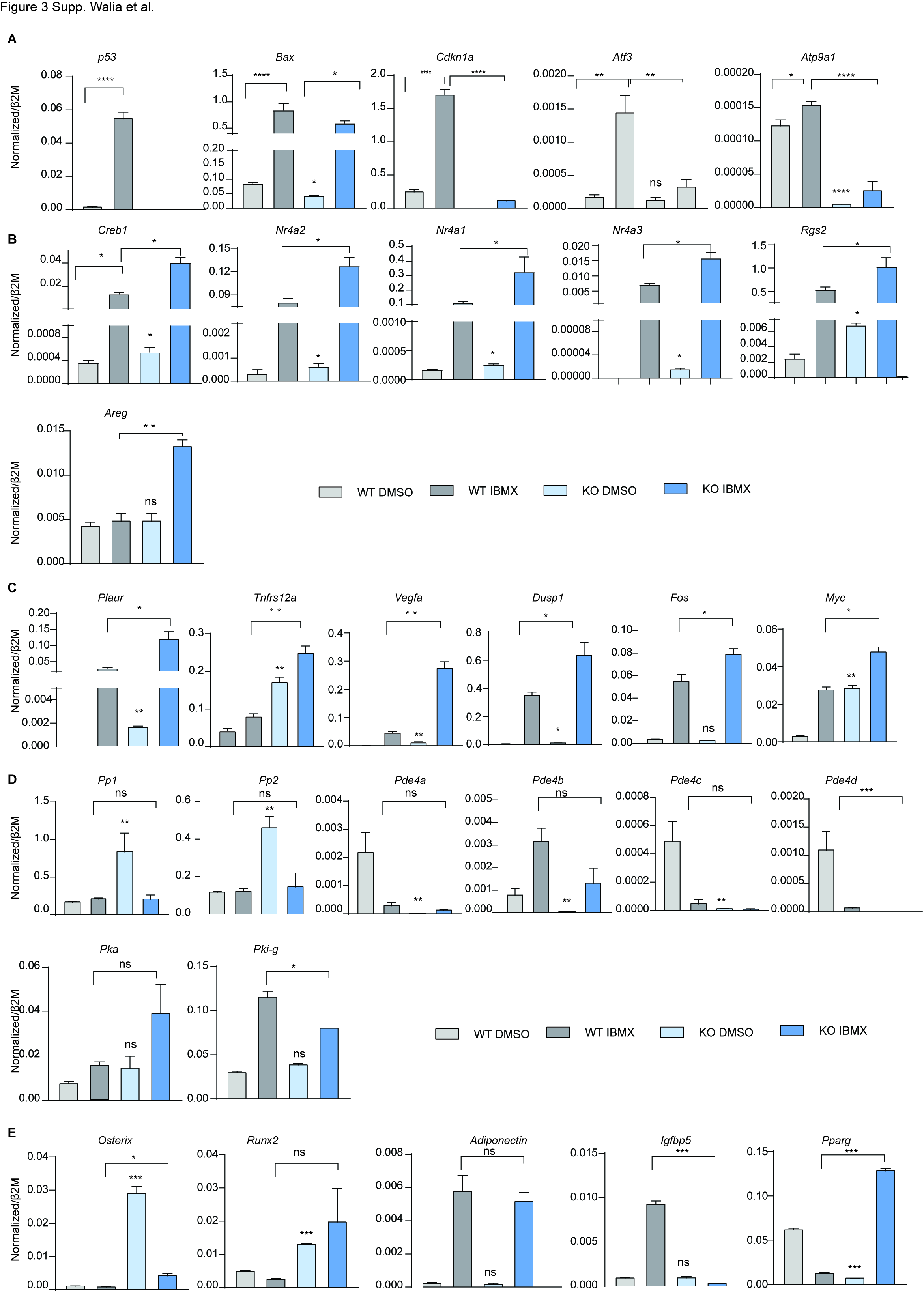

Supplement: Supplementary file 3 — Supplemental Figure 3 [file 41419_2018_944_MOESM3_ESM.tif]

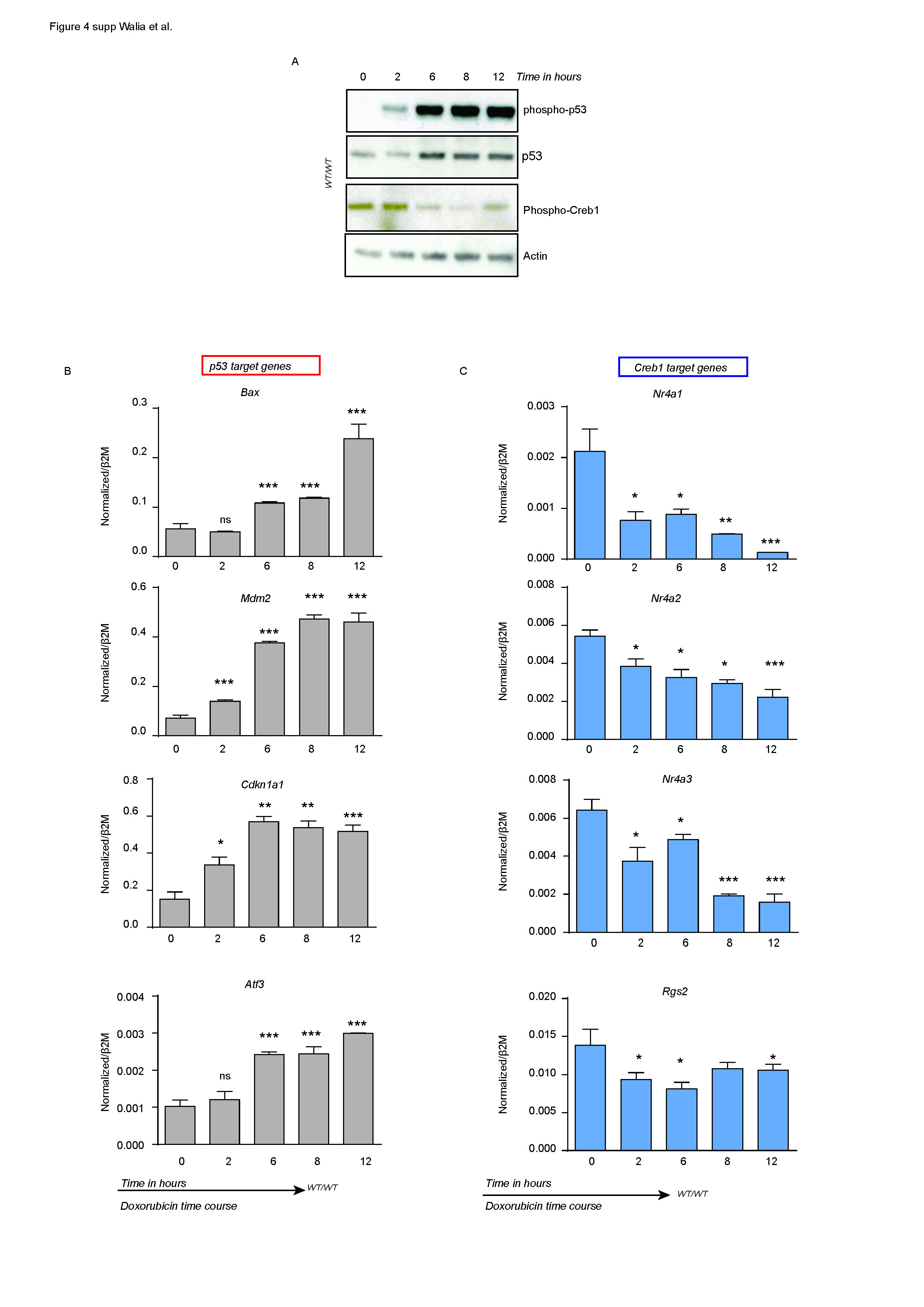

Supplement: Supplementary file 4 — Supplemental Figure 4 [file 41419_2018_944_MOESM4_ESM.jpg]

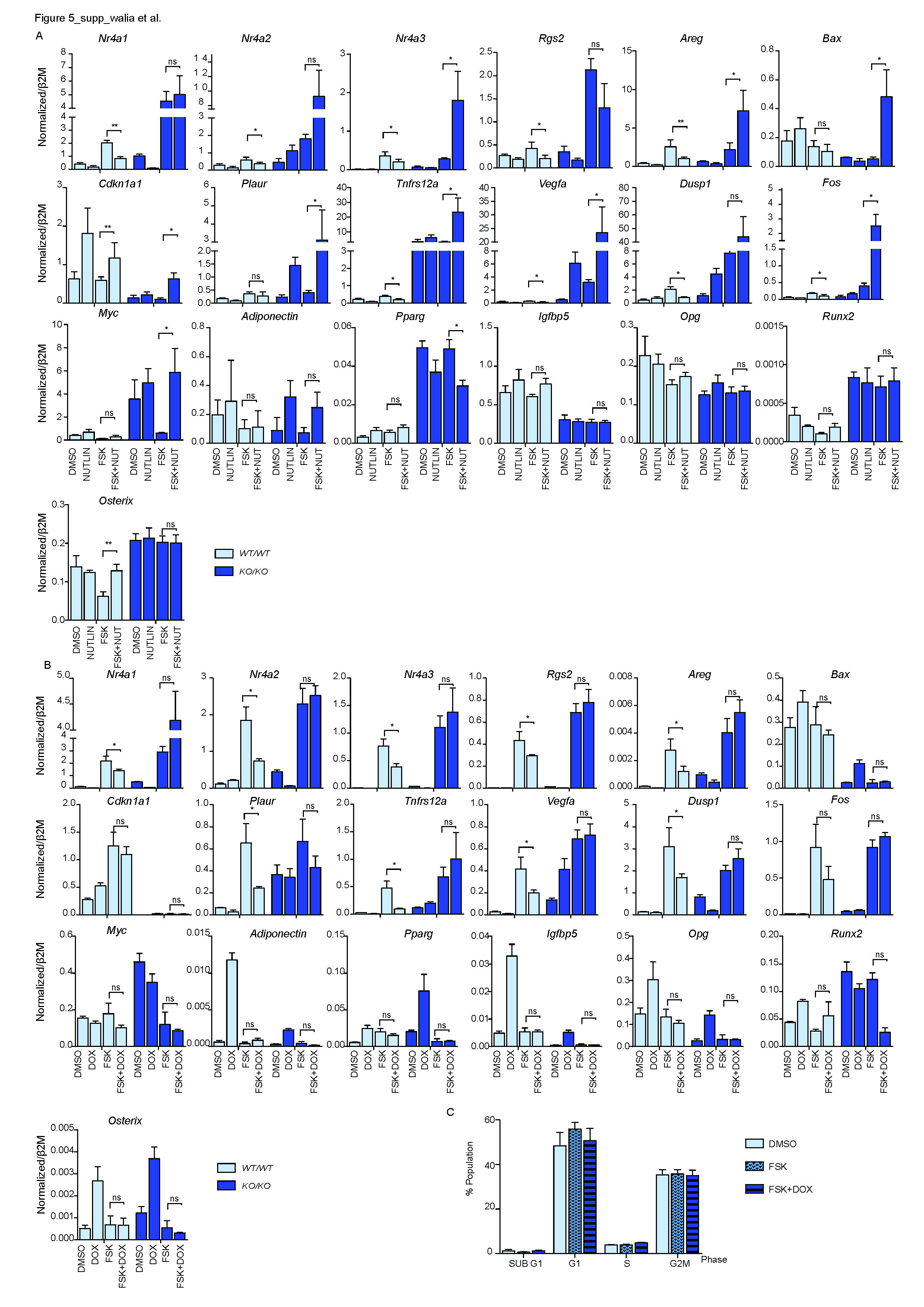

Supplement: Supplementary file 5 — Supplemental Figure 5 [file 41419_2018_944_MOESM5_ESM.jpg]
