# Supplementary material for: Tolerance to sustained activation of the cAMP/Creb pathway activity in osteoblastic cells is enabled by loss of p53
Source: Cell Death Dis. 2018 Aug 28;9(9):844. doi: 10.1038/s41419-018-0944-8 (PMC6113249; doi:10.1038/s41419-018-0944-8)
Supplement: Supplementary file 6 — Supplementary figure legends [file 41419_2018_944_MOESM6_ESM.doc]

**Supplemental Figure Legends**

**Supplemental Figure 1. Loss of p53 in primary osteoblasts leads to accelerated differentiation and increased PTHrP-cAMP-Creb1 axis activity**. **(A)** mRNA for osteogenic differentiation markers at the indicated days following *in vitro* differentiation of isogenic p53*^WT/WT^* and p53*^KO/KO^* cells (mean±SEM (n=3). **(B)** mRNA for adipogenic differentiation markers at the indicated days following *in vitro* differentiation of isogenic p53*^WT/WT^* and p53*^KO/KO^* cells (mean ± SEM, n=3). (**C-D**) Western blot of p53, pCREB1 and Creb1, β-Actin used as a loading control. Data representative of 2 independent cell lines from each. For all panels: **P*<0.05, ***P*<0.01, ****P*<0.001.

**Supplemental Figure 2. cAMP-induced apoptosis in normal osteoblasts is mediated by p53**. (**A**) cAMP accumulation in p53*^WT/WT^* and p53*^KO/KO^*, forskolin treatment for 15 mins (IBMX 100μM) . (**B**) mRNA for *P53, CDKN1A1, ATP9A1, MDM2* by qPCR; Expression levels normalized to *β2m*; mean±SEM, n=2 in HEK293T cells. (**C**) mRNA for *CREB1, NR4A1, NR4A2, NR4A3* by qPCR; Expression levels normalized to *β2m*; mean±SEM, n=2 in HEK293T cells. Data pooled from 2 independent cultures; mean±SEM. For all panels: **P*<0.05, ***P*<0.01, ****P*<0.001.

**Supplemental Figure 3. cAMP elevation in p53*^KO/KO^* cells activates pro-proliferative gene expression signatures**. (**A**) qPCR data of p53 target genes between indicated cell types; 3 independent isogenic cell lines for each, expressed as mRNA normalized to *β2m.* **(B)** qPCR data of Creb1 target genes between indicated cell types; 3 independent isogenic cell lines for each, expressed as mRNA normalized to *β2m.* **(C)** qPCR data for expression of genes important for cellular proliferation between indicated cell types; 3 independent isogenic cell lines for each, expressed as mRNA normalized to *β2m.* **(D)** qPCR data for genes that are negative regulators of cAMP. Expression of the genes between indicated cell types; 3 independent isogenic cell lines for each, expressed as mRNA normalized to *β2m.* **(E)** qPCR data for genes important in development. Expression of the genes between indicated cell types; 3 independent isogenic cell lines for each, expressed as relative expression normalized to *β2m.* For all panels: **P*<0.05, ***P*<0.01, ****P*<0.001.

**Supplemental Figure 4. Activation of p53 directly suppresses Creb1 function. (A)** Western blot in p53*^WT/WT^* cells of p53, pCreb1 and Creb1; β-Actin as loading control in primary osteoblast. (**B**) qPCR validation of p53 target gene expression following 500nM doxorubicin treatment over time course of 12 hours; 3 independent isogenic p53*^WT/WT^* cells mean±SEM, n=3. **(C)** qPCR validation of Creb1 target gene expression following 500nM doxorubicin treatment over time course of 12 hours; 3 independent isogenic p53*^WT/WT^* cells mean± SEM, n=3.

**Supplemental Figure 5. Activation of p53 and accumulation of cAMP leads to increased apoptosis. (A)** qPCR data for the expression of p53 and Creb1/pro-proliferative targets, developmental targets between cell types post co-treatment with forskolin and Nutlin-3a; Data from 3 independent isogenic cell lines for each, expressed as relative expression normalized to *β2m*. Data expressed as mean±SEM (n=3). **(B)** qPCR data for the expression of p53 and Creb1/pro-proliferative targets, developmental targets between cell types post co-treatment with forskolin and doxorubicin; Data from 3 independent isogenic cell lines for each, expressed as relative expression normalized to *β2m*. Data expressed as mean±SEM (n=3). **(C)** Cell cycle analysis of p53*^WT/WT^* cells treated with DMSO, doxorubicin or doxorubicin and forskolin. For all panels: **P*<0.05, ***P*<0.01, ****P*<0.001. For all panels: **P*<0.05, ***P*<0.01, ****P*<0.001.
