# Supplementary material for: Tolerance to sustained activation of the cAMP/Creb pathway activity in osteoblastic cells is enabled by loss of p53
Source: Cell Death Dis. 2018 Aug 28;9(9):844. doi: 10.1038/s41419-018-0944-8 (PMC6113249; doi:10.1038/s41419-018-0944-8)
Supplement: Supplementary file 7 — Supplementary Table [file 41419_2018_944_MOESM7_ESM.doc]

| *Gene* | Forward Primer | Reverse Primer |
| --- | --- | --- |
| *Osterix* | cctctcccttctccctctc | ctggagccatagtgagcttc |
| *Runx2* | gctattaaagtgacagtggacg | ggcgatcagagaacaaactagg |
| *Pthr1* | ccccgagtctaaagagaacaag | gtaatcgggacaaggtactgc |
| *Pthrp* | GATTCCTACACAAGTCCCCAG | GAATACCAGGACACTCCACTG |
| *Osteocalcin* | accatctttctgctcactctg | gttcactaccttattgccctcc |
| *Sost* | acaaccagaccatgaaccg | caggaagcgggtgtagtg |
| *Pparg* | GAACCTTCTAACTCCCTCATGG | TCAATCGGATGGTTCTTCGG |
| *Adiponectin* | TGTCTGTACGATTGTCAGTGG | GCAGGATTAAGAGGAACAGGAG |
| *P53* | CCCAGGATGTTGAGGAGTTTT | TTTTGAGAAGGGACAAAAGATGA |
| *Bax* | TTTGCTTCAGGGTTTCATCC | CACTCGCTCAGCTTCTTGGT |
| *Cdkn1a1* | GACAAGAGGCCCAGTACTTCC | GATAGAAATCTGTCAGGCTGGTCT |
| *Atf3* | CTGAAGAATGAGAAACAGCATTTG | CAATGTTCCTTCTTTTATCTGTTGG |
| *Atp9a1* | TTGTTCCCGAAATGAGACTTG | TAGCATCGGATCTCCTCCAC |
| *Creb1* | CAAGTCCAAACAGTTCAGATTTCA | TGGTGCATCAGAAGATAAGTCATT |
| *Nr4a1* | CTCCTCCACGTCTTCTTCCTC | CAGGGACTGCCATAGTACTCAGA |
| *Nr4a2* | ACTGAAATTACTGCCACCACTTCT | TGTGCATCTGAATGTCTTCTACCT |
| *Nr4a3* | GGTGCAGAAAAATGCAAAATATG | CTGTCTGTACGCACAACTTCCTTA |
| *Rgs2* | GTCCTCAAAAGCAAGGAAAATCTA | CATCAAACTGTACACCCTCTTCTG |
| *Areg* | CACAGGGGACTACGACTACTCAG | TCTTCCTTTTGGGTTTTTCTGTAG |
| *Plaur* | ACAGAGCACTGTATTGAAGTGGTG | GAAAGGTCTGGTTGCTATGGAA |
| *Tnfrs12a* | GCTGGTTTCTAGTTTCCTGGTCT | GTCTCCTCTATGGGGGTAGTAAACTT |
| *Vegfa* | GAAACCATGAACTTTCTGCTCTCT | ACTTGATCACTTCATGGGACTTCT |
| *Dusp1* | TCACGCTTCTCGGAAGGATA | TGATGTCTGCCTTGTGGTTG |
| *Fos* | GCTATCTCCTGAAGAGGAAGAGAAA | AACGCAGACTTCTCATCTTCAAGT |
| *Myc* | AGG AGA CAC CGC CCA CCA CC | TGC TGT GGC CTC GGG ATG GA |
| *Pp1* | ATACCCGGAGAACTTCTTTCTTCTA | AGCAGCTATAGGCAGACAGTTAAAA |
| *Pp2* | ACTATTTGTTTCTCGGGGACTATGT | CACTCATCATAAAATCCGTAGATCC |
| *Pde4a* | GTTGACTTCTTCTGCGAGACCT | CTAGGAACGTGTTGGAAATGTACTC |
| *Pde4b* | AAAAGAATTTACTGCTTCGGAATCT | ACTTTCTGAAAAAGCATGGTGAAT |
| *Pde4c* | GGTCTCCTAGACGCTCAGTCC | ACAATCATGTCTTCTCCATGTAGGT |
| *Pde4d* | GAGGGACACTTGTGATGTACTCTCT | AGTCTGCTTGTTCCAACTGTCTG |
| *Pka* | GCGGTTCCCATCCCACTT | GTGAGATCCACCTGCAGAAGGT |
| *Pkig* | CCTGACATCCAGGGTGACTC | CTCTGCCTGTCCTTCTGCTC |
